# The phosphorylated form of FTY720 activates PP2A, represses inflammation and is devoid of S1P agonism in A549 lung epithelial cells

**DOI:** 10.1038/srep37297

**Published:** 2016-11-16

**Authors:** Md. Mostafizur Rahman, Laura Prünte, Leonard F. Lebender, Brijeshkumar S. Patel, Ingrid Gelissen, Philip M. Hansbro, Jonathan C. Morris, Andrew R. Clark, Nicole M. Verrills, Alaina J. Ammit

**Affiliations:** 1 Faculty of Pharmacy, University of Sydney, NSW, 2006, Australia; 2 Priority Research Centre for Healthy Lungs, Hunter Medical Research Institute and the University of Newcastle, NSW, 2308, Australia; 3 School of Chemistry, University of NSW, NSW, 2052, Australia; 4 Institute of Inflammation and Ageing, College of Medical and Dental Sciences, University of Birmingham, Edgbaston B15 2TT, United Kingdom; 5 School of Biomedical Sciences and Pharmacy, Faculty of Health and Medicine, University of Newcastle, NSW, 2308, Australia; 6 Woolcock Emphysema Centre, Woolcock Institute of Medical Research, University of Sydney, NSW, Australia; 7 School of Life Sciences, Faculty of Science, University of Technology Sydney, NSW, Australia

## Abstract

Protein phosphatase 2A (PP2A) activity can be enhanced pharmacologically by PP2A-activating drugs (PADs). The sphingosine analog FTY720 is the best known PAD and we have shown that FTY720 represses production of pro-inflammatory cytokines responsible for respiratory disease pathogenesis. Whether its phosphorylated form, FTY720-P, also enhances PP2A activity independently of the sphingosine 1-phosphate (S1P) pathway was unknown. Herein, we show that FTY720-P enhances TNF-induced PP2A phosphatase activity and significantly represses TNF-induced interleukin 6 (IL-6) and IL-8 mRNA expression and protein secretion from A549 lung epithelial cells. Comparing FTY720 and FTY720-P with S1P, we show that unlike S1P, the sphingosine analogs do not induce cytokine production on their own. In fact, FTY720 and FTY720-P significantly repress S1P-induced IL-6 and IL-8 production. We then examined their impact on expression of cyclooxygenase 2 (COX-2) and resultant prostaglandin E_2_ (PGE_2)_ production. S1P did not increase production of this pro-inflammatory enzyme because COX-2 mRNA gene expression is NF-κB-dependent, and unlike TNF, S1P did not activate NF-κB. However, TNF-induced COX-2 mRNA expression and PGE_2_ secretion is repressed by FTY720 and FTY720-P. Hence, FTY720-P enhances PP2A activity and that PADs can repress production of pro-inflammatory cytokines and enzymes in A549 lung epithelial cells in a manner devoid of S1P agonism.

Protein phosphatase 2A (PP2A) is a ubiquitously expressed serine/threonine phosphatase and a master regulator of inflammatory cell signaling. PP2A is an important target for anti-cancer therapy^1^ and has recently emerged as playing an important role in chronic respiratory diseases, notably asthma. In *in vivo* mouse models of allergic airway disease, PP2A activators were shown to repress respiratory inflammation^2^,^3^. PP2A levels (and therefore activity) were reduced in these models of asthma^2^ and PP2A activity could be reinstated pharmacologically to repress hallmark features of allergic and rhinovirus-induced airway disease^2^,^3^. *In vitro*, we have shown that PP2A activators inhibit cytokine production in a cellular model of airway inflammation using A549 lung epithelial cells stimulated with tumor necrosis factor (TNF) *in vitro*^4^,^5^.

PP2A is a trimolecular complex and a druggable target that can be activated pharmacologically^1^. PP2A-activating drugs (now collectively referred to as PADs) have emerged as novel therapeutics in cancer^6^ and offer demonstrable potential as novel anti-inflammatories in chronic respiratory disease^2^,^3^,^4^,^5^. The best known PAD is the sphingosine analog FTY720 (Fingolimod). Although the molecular mechanisms responsible for the beneficial actions of PADs in disease have begun to be revealed^7^, many questions remain.

In this study we investigated the impact of phosphorylation of FTY720 on the capacity of this sphingosine analog to enhance PP2A activation. Moreover, we directly examine whether FTY720-P, being an agonist/functional antagonist of the S1P pathway, has pro-inflammatory effects in a cellular model of airway inflammation *in vitro*. This is an important consideration for future development of PADs based on the sphingosine backbone into the respiratory clinic. This is because we discovered that S1P is elevated in asthma^8^ and this molecule has been shown to drive the development of a pro-inflammatory phenotype^8^,^9^,^10^,^11^. Herein, we show that like FTY720, FTY720-P increases TNF-induced PP2A activity and represses the production of pro-inflammatory cytokines interleukin 6 (IL-6) and IL-8 and the enzyme cyclo-oxygenase 2 (COX-2). Notably, FTY720 and FTY720-P are devoid of S1P-pathway agonism in A549 lung epithelial cells.

## Results

### FTY720-P enhances TNF-induced PP2A phosphatase activity in A549 cells

**Figure 1:**
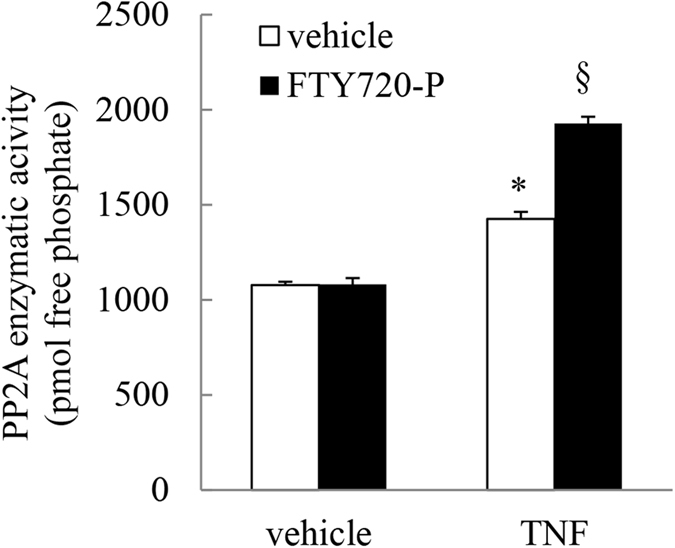
FTY720-P enhances TNF-induced PP2A phosphatase activity in A549 cells. A549 cells were pretreated with 2.5 μM FTY720-P for 6 h, compared to vehicle-treated controls. Cells were treated with vehicle or TNF (4 ng/ml) and then PP2A enzymatic activity (measured as pmol free phosphate) detected at 1 h. Statistical analysis was performed using one-way ANOVA then Bonferroni’s post-test (where * denotes a significant effect of TNF compared to vehicle-treated cells, and § denotes a significant effect of FTY720-P on TNF-induced PP2A activity (*P* < 0.05)). Data are mean + SEM values from n = 3 independent experiments.

The classical PP2A activator is FTY720 and we have previously published that FTY720 significantly increases TNF-induced PP2A activity in A549 cells^5^. In the current study we were interested in examining the impact of the phosphorylated form of FTY720, i.e. FTY720-P, on PP2A enzymatic activity. There was no significant effect of FTY720-P when A549 cells were pretreated with FTY720-P (2.5 μM) alone; the PP2A enzymatic activity in FTY720-P-treated cells was not significantly different from cells treated with vehicle (Fig. 1). In contrast, FTY720-P significantly enhanced PP2A activity induced by 4 ng/ml TNF (*P* < 0.05).

### FTY720-P significantly represses TNF-induced IL-6 and IL-8 mRNA expression and protein secretion

**Figure 2:**
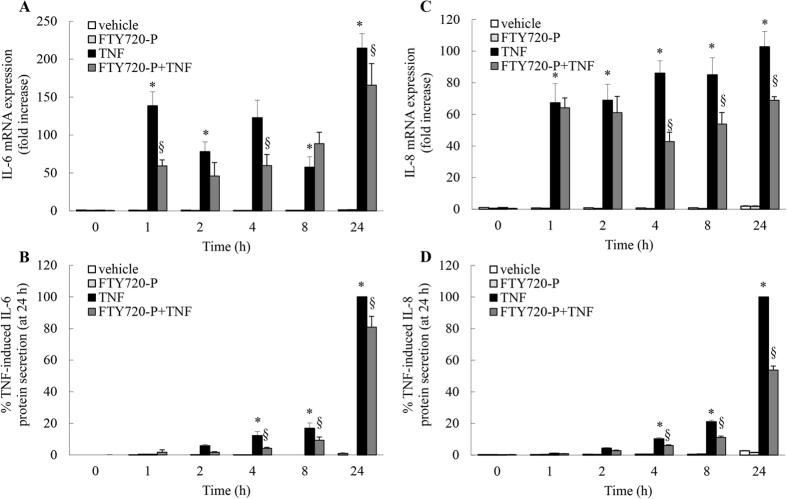
FTY720-P significantly represses TNF-induced IL-6 and IL-8 mRNA expression and protein secretion. A549 cells were pretreated with 2.5 μM FTY720-P for 6 h, compared to vehicle-treated controls. Cells were treated with vehicle or TNF (4 ng/ml) and then (**A**,**C**) IL-6 and IL-8 mRNA expression (results expressed as fold increase compared to vehicle-treated cells at 0 h) and (**B**,**D**) IL-6 and IL-8 protein secretion measured (results expressed as a percentage of TNF-induced cytokine secretion at 24 h: i.e. (**B**) 1,769.0 ± 517.6 pg/ml and (**D**) 56,254.8 ± 805.2 pg/ml) at the indicated times. Statistical analysis was performed using two-way ANOVA then Bonferroni’s post-test (where * denotes a significant effect of TNF compared to vehicle-treated cells, and **§** denotes significant repression of TNF-induced cytokine production by FTY720-P (*P* < 0.05)). Data are mean + SEM values from n = 4 independent experiments.

Our recent studies have underscored the anti-inflammatory impact of PP2A activation on respiratory inflammation *in vitro*^4^,^5^,^12^. Both well-known (FTY720^4^,^5^): and more recently-reported (AAL(S)^4^,^5^; theophylline^12^) molecules that increase PP2A enzymatic activity can significantly repress cytokine production *in vitro*. Thus, it follows that if FTY720-P increases TNF-induced PP2A enzymatic activity, then it should inhibit cytokine mRNA expression and protein secretion. To test this, we utilized a cellular model of airway inflammation using A549 lung epithelial cells stimulated with TNF *in vitro*^5^,^13^. In agreement with our earlier study^5^, TNF significantly increased IL-6 and IL-8 mRNA expression and protein secretion (Fig. 2: *P* < 0.05). Cells pretreated with FTY720-P significantly repressed TNF-induced IL-6 mRNA expression (notably at 1 and 4 h) and this resulted in significantly less IL-6 protein secretion from A549 cells from 4–24 h (Fig. 2A and B: *P* < 0.05). TNF-induced IL-8 mRNA expression and protein secretion was also significantly repressed by FTY720-P (Fig. 2C: *P* < 0.05). This resulted in 46.2 ± 2.5% inhibition of TNF-induced IL-8 protein secretion from A549 cells at 24 h (Fig. 2D: *P* < 0.05). Thus, like FTY720^5^, FTY720-P significantly represses TNF-induced IL-6 and IL-8 mRNA expression and protein secretion.

### FTY720 and FTY720-P significantly repress S1P-induced IL-6 and IL-8 mRNA expression and protein secretion

**Figure 3:**
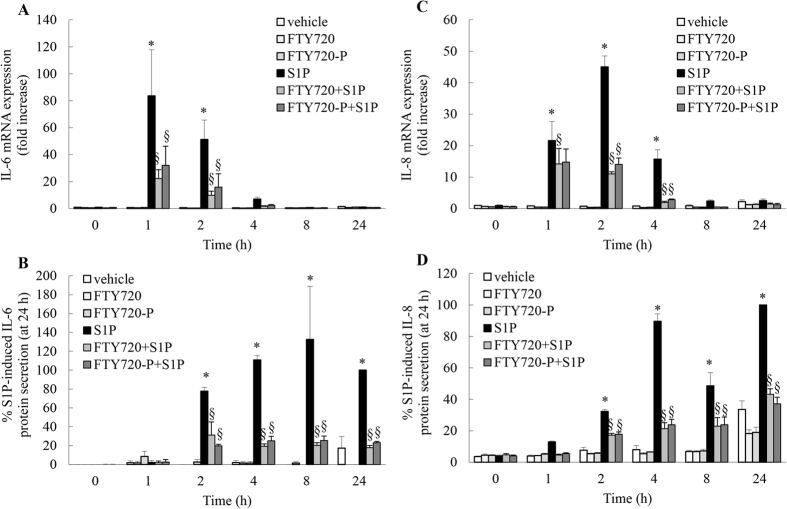
FTY720 and FTY720-P significantly repress S1P-induced IL-6 and IL-8 mRNA expression and protein secretion. A549 cells were pretreated with 2.5 μM FTY720 or FTY720-P for 6 h, compared to vehicle-treated controls. Cells were treated with vehicle or S1P (1 μM) and then (**A**,**C**) IL-6 and IL-8 mRNA expression (results expressed as fold increase compared to vehicle-treated cells at 0 h) and (**B**,**D**) IL-6 and IL-8 protein secretion measured (results expressed as a percentage of TNF-induced cytokine secretion at 24 h: i.e. (**B**) 100.5 ± 34.1 pg/ml and (**D**) 4,363.0 ± 241.1 pg/ml) at the indicated times. Statistical analysis was performed using two-way ANOVA then Bonferroni’s post-test (where * denotes a significant effect of S1P compared to vehicle-treated cells, and **§** denotes significant repression of S1P-induced cytokine production by FTY720-P (*P* < 0.05)). Data are mean + SEM values from n = 3 independent experiments.

To extend these studies further we examined whether cytokine production induced by another clinically important mediator in asthmatic inflammation – S1P – was repressed by FTY720 and FTY720-P. A549 cells were stimulated with S1P (1 μM) for 0–24 h and resultant IL-6 and IL-8 mRNA expression and protein secretion were assessed (Fig. 3). Our study is the first to demonstrate that S1P significantly induces IL-6 mRNA expression and protein secretion in A549 cells (Fig. 3A and B: *P* < 0.05). Milara *et al.* have previously reported that S1P induces IL-8 mRNA expression and protein secretion from A549 cells^14^, and our results shown in Fig. 3C and D concur. Notably, both FTY720 and its phosphorylated form FTY720-P robustly repress S1P-induced IL-6 and IL-8 mRNA expression and protein secretion from A549 cells (Fig. 3: *P* < 0.05).

### S1P does not induce COX-2 mRNA expression, or PGE_2_ secretion

**Figure 4:**
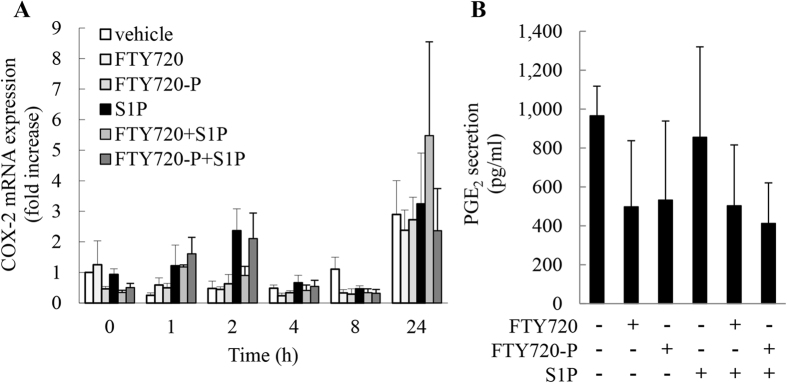
S1P does not induce COX-2 mRNA expression, or PGE_2_ secretion. A549 cells were pretreated with 2.5 μM FTY720 or FTY720-P for 6 h, compared to vehicle-treated controls. Cells were treated with vehicle or S1P (1 μM) and then (**A**) COX-2 mRNA expression measured at the indicated times (results expressed as fold increase compared to vehicle-treated cells at 0 h) and (**B**) secreted PGE_2_ at 24 h measured by enzyme immunoassay. Data are mean + SEM values from n = 3 independent experiments.

In structural cell types apart from A549 lung epithelial cells, S1P has been shown by us^15^ and others^16^,^17^,^18^,^19^ to increase production of the pro-inflammatory enzyme cyclo-oxygenase 2 (COX-2) and result in increased secretion of prostaglandin E_2_ (PGE_2_), a key prostanoid with multi-functional actions in airway inflammation^20^. We were intrigued to examine whether COX-2 mRNA expression and PGE_2_ secretion was induced by S1P in A549 cells. We also examined the effect of FTY720 and FTY720-P in parallel. S1P did not result in a significant induction of COX-2 mRNA at any time point measured (Fig. 4A). Nor was PGE_2_ secretion at 24 h increased by S1P (Fig. 4B).

### TNF-induced, NF-κB-dependent COX-2 mRNA expression and PGE_2_ secretion can be repressed by FTY720 and FTY720-P

**Figure 5:**
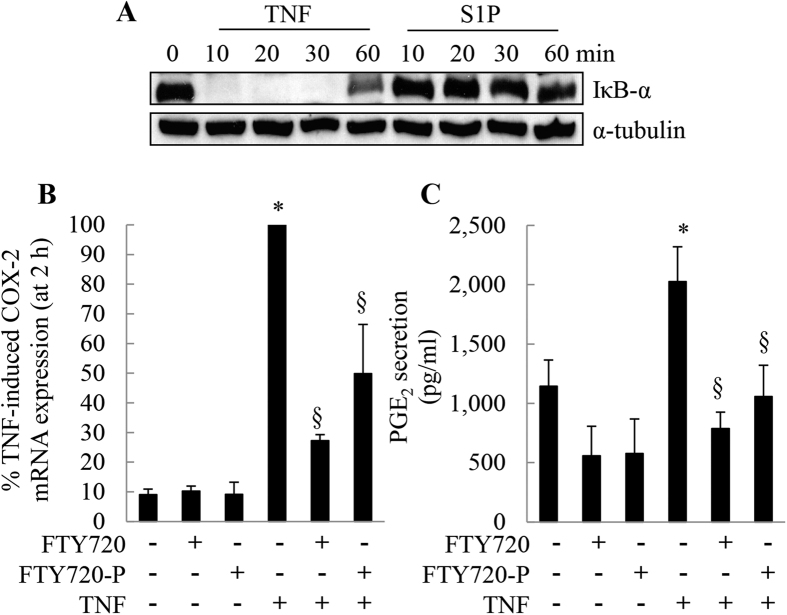
TNF-induced, NF-κB-dependent COX-2 mRNA expression and PGE_2_ secretion can be repressed by FTY720 and FTY720-P. (**A**) To demonstrate that unlike S1P, TNF induced IκB-α degradation (a surrogate measure of NF-κB activation), A549 cells were stimulated with TNF (4 ng/ml) or S1P (1 μM) for 0, 10, 30, and 60 min. Cells were then lysed and IκB-α analyzed by Western blotting, compared to α-tubulin as a loading control. (**B**,**C**) A549 cells were pretreated with 2.5 μM FTY720 or FTY720-P for 6 h, compared to vehicle-treated controls. Cells were treated with vehicle or TNF (4 ng/ml) and then (**B**) COX-2 mRNA expression measured (results expressed as % TNF-induced COX-2 mRNA expression (at 2 h)) and (**C**) secreted PGE_2_ at 24 h measured by enzyme immunoassay. Statistical analysis was performed using the Student’s unpaired *t* test (where * denotes a significant effect of TNF compared to vehicle-treated cells, and **§** denotes significant repression by FTY720 or FTY720-P (*P* < 0.05)). Data are mean + SEM values from (**A**) n = 3 and (**B**,**C**) n = 4 independent experiments.

COX-2 is an inducible enzyme that is regulated at multiple levels (as reviewed in ref. 20). One of the major transcriptional regulators of COX-2 gene expression is nuclear factor kappa B (NF-κB). The 5′-untranslated region of the COX-2 gene contains consensus binding sites for a number of *trans*-regulatory transcription factors, including two NF-κB sites^21^. Given that S1P did not induce COX-2 mRNA expression in A549 cells, the possibility exists that S1P does not activate NF-κB in this cell type. To test this, we examined the degradation of IκB-α after stimulation with S1P, compared to TNF as a positive control^22^. IκB-α degradation is a commonly-used surrogate measure of NF-κB activation^22^. In unstimulated cells, IκB-α forms a complex with NF-κB that renders it transcriptionally inactive. If IκB-α is degraded upon cell stimulation, NF-κB is released from the inhibitory complex and NF-κB-dependent transcriptional regulation can ensue. We found that TNF induces IκB-α degradation in A549 cells (Fig. 5A). The high levels of IκB-α observed at 0 min were rapidly degraded after 10 min stimulation with TNF. This degradation is sustained for up to 30 min but recovers by 60 min. This temporal profile is characteristic of TNF-induced NF-κB activation kinetics previously demonstrated by us in primary airway smooth muscle cells^22^. In contrast, the levels of IκB-α was unchanged by S1P treatment (Fig. 5A). Given that TNF activates NF-κB, and that COX-2 gene expression is NF-κB-dependent, we assessed the impact of treatment on these events. We show that TNF induces COX-2 mRNA expression and this can be repressed by FTY720 and FTY720-P (Fig. 5B: *P* < 0.05). Finally, we demonstrate that TNF significantly increases PGE_2_ secretion at 24 h (Fig. 5C: *P* < 0.05) and this too can be repressed by FTY720 and FTY720-P.

## Discussion

Although FTY720 is well-established as a PAD, less is known about the PP2A-activating function of its phosphorylated form - FTY720-P – or its ability to repress cytokines that drive respiratory inflammation or disease. We address this herein, using a cellular model of airway inflammation and show that FTY720-P increases PP2A enzymatic activity in A549 lung epithelial cells in a manner comparable to that recently published for the non-phosphorylated FTY720^4^,^5^. Similar to other molecules with demonstrated PP2A-activating capacity (including AAL(S)^5^ and theophylline^12^), FTY720-P significantly repressed the production of the pro-inflammatory cytokines IL-6 and IL-8. Moreover, both FTY720 and FTY720-P repressed TNF-induced COX-2 mRNA expression and PGE_2_ secretion. Notably, this repression was exerted without impacting on the pro-inflammatory S1P pathway, as the phospho- and non-phospho-form of the sphingosine analog FTY720 did not increase cytokine production alone.

FTY720 (fingolimod) is used clinically for the treatment of multiple sclerosis (MS). The way in which FTY720 achieves its beneficial action in MS is via repression of S1P-regulated lymphocyte egress^23^. The nature of FTY720’s inhibitory interaction with the S1P/S1P receptor (S1PR) system can be classified as functional agonism/antagonism. In brief, FTY720 is internalized by cells and phosphorylated by the enzyme sphingosine kinase 2 to phosphorylated form (FTY720-P). FYT720-P resembles the naturally-occurring ligand S1P; thus, when FTY720-P leaves the cell and it then activates one of five S1P receptors – i.e. S1PR1, causing this receptor to becomes downregulated. Hence the cognate receptor is unavailable for subsequent stimulation by the naturally-occurring ligand, S1P^23^. An understanding of this mechanism of drug action is a central consideration in designing better PADs, especially those that are sphingosine analogs (like FTY720), because it is imperative to ensure that they are devoid of interaction with the S1P pro-inflammatory pathway. This is particularly important given that S1P is a driver of respiratory disease pathogenesis^11^ and we have found elevated levels of S1P in the bronchoalveolar lavage of people with asthma, compared to normal volunteers^8^. There are two key points to consider here: firstly, FTY720-P resembles S1P; secondly, FTY720-P has to activate the S1P receptor. Thus it follows that if the actions of FTY720-P occurred via the S1P pathway, then they should resemble the actions of S1P. We have tested this in the current study and show that S1P significantly increases IL-8 and IL-6 mRNA and protein secretion. In contrast, neither FTY720 nor FTY720-P do not. These data support the assertion that the effects of FTY720/FTY720-P are independent of the S1P/S1P receptor-cell signaling pathway. As a further proof, the repressive impact of FTY720-P/FTY720-P mimics those of AAL(S), a sphingosine analog that has been chemically modified so that it is not phosphorylatable by sphingosine kinase 2 and thus is devoid of S1P agonism^5^.

The current study extends our understanding of the repressive actions of FTY720 in the context of respiratory inflammation measured *in vitro* and shows that in addition to inhibition of IL-6 and IL-8^4^,^5^, FTY720 also represses the expression of the COX-2 gene and production of an important prostanoid, PGE_2_. Moreover, these pro-inflammatory cytokines and enzyme are also repressed by the phospho-form of FTY720. It is notable that the FTY720-P on IL-6 induction is comparatively modest. These data are similar to our prior publications demonstrating the differential impact of FTY720 on IL-6 and IL-8^4^,^5^ and align with our previous studies elucidating the signaling mechanisms responsible for IL-6 *versus* IL-8 induction. In A549 lung epithelial cells^4^ and airway smooth muscle cells^24^,^25^, we have reported that TNF-induced IL-6 production is p38 MAPK-driven, while IL-8 expression is due is both ERK- and p38 MAPK-acting in concert. Since two phosphoproteins may be reversed by PP2A activation in the case of IL-8, compared to one (i.e. p38 MAPK) for IL-6, we suggest that the inhibitory impact of FTY720-P on IL-8 is greater than IL-6.

Notably, we show for the first time that FTY720-P increases PP2A enzymatic activity in A549 cells. Taken together, these studies extend our understanding that a broad range of compounds can induce PP2A activation under experimental conditions mimicking inflammation. Although FTY720, FTY720-P and AAL(S) are all sphingosine analogs, forskolin^26^, theophylline^12^ and salmeterol^3^ are structurally distinct and thus argue against a common pharmacophoric model. The molecular mechanisms responsible for PP2A activation in the context of respiratory inflammation are unknown at present. PP2A exists as a tri-molecular complex of a catalytic subunit (C), a structural subunit (A), and a variable regulatory subunit (B) of which there are at least 3 different families (B55, B56, B″) each with several isoforms^27^. Given that PP2A exerts precise and specific regulation of multiple signaling pathways in a co-ordinated manner it is not surprising that PP2A is highly regulated at multiple levels^28^. These include transcriptional and post-transcriptional regulation, control by post-translational modifications and ubiquitin-mediated proteasomal degradation, as well as temporal and sub-cellular control^28^,^29^. Moreover, PP2A enzymatic activity is regulated by endogenous inhibitory proteins (including ANP32A^29^, ANP32C^30^, I2PP2A/SET^31^, and CIP2A^32^) and modulated by the nature and identity of the PP2A regulatory subunits. Whether interaction of PADs with PP2A-inhibitory interactors (such as reported for FTY720 and I2PP2A/SET in the regulation of tumor suppression^7^) is responsible for their beneficial actions in respiratory inflammation is unknown at present and warrants further in-depth investigation.

However, the identification of FTY720-P as a PAD does not appear to readily align with the current view from the literature. Indeed, a number of published reports have stated that FTY720-P does not activate PP2A^1^,^33^,^34^. However, apart from the cell type differences, there is an important experimental distinction that should be highlighted. That is, we have only shown increased PP2A activation with any of these compounds when cells have been stimulated, and thus far all the molecules that we have shown increase TNF-induced PP2A activity^4^,^5^, i.e. FTY720, AAL(S), theophylline, and FTY720-P, do not affect basal PP2A enzymatic activity. In contrast, the studies performed in the context of tumorigenesis have attempted to restore and reactivate low levels of PP2A activity that are functionally important for malignancy^1^,^34^. Why FTY720-P activates PP2A in A549 cells not at baseline but only when the cells were stimulated with TNF is unknown at present. PP2A regulation is complex and given that enzymatic activity can be modulated by the nature and identity of PP2A subunits and is regulated by endogenous inhibitory proteins, myriad potential explanations exist. It is possible that an inflammatory stimulus modulates PP2A isoforms or the regulatory subunits. Additionally, whether another phosphatase is responsible is also plausible; e.g. TNF (or S1P) may induce a phosphatase that dephosphorylates FTY720-P. An in depth investigation of PP2A regulation is required to fully elucidate the underlying molecular mechanisms.

A limitation of our study is that we have not shown that the effects of FTY720-P on cytokine production are the direct result of PP2A activation. Although it is possible to knock-down individual components of PP2A (for example, PP2A-C), redundancy and compensatory mechanisms exist that make the proposed research methodology problematic. The fact that remains that FTY720-P could exert anti-inflammatory effects in a PP2A-independent manner. Moreover, our study has not identified the downstream effectors responsible for the repressive effects of FTY720-P on cytokines. Our previous study showed that the molecule responsible for PP2A repressive actions is the mRNA destabilizing protein tristetraprolin (TTP). In Rahman *et al.*^5^, we showed that activating PP2A with FTY720 enhanced TTP anti-inflammatory function in A549 lung epithelial cells. FTY720-P may act via a PP2A/TTP-dependent pathway, although it is important to note that PP2A is a ubiquitously expressed serine/threonine phosphatase responsible for dephosphorylation of TTP^35^, amongst other proteins^32^,^36^,^37^.

In summary, the phosphorylated form of FTY720 (FTY720-P) joins the list of small molecule activators of PP2A (collectively referred to as PADs). FTY720-P represses inflammation and does so in a manner that is devoid of agonism of the S1P-mediated cellular signaling pathway. Thus, small molecule activators of PP2A offer exciting potential as novel anti-inflammatories in chronic respiratory diseases.

## Methods

### Chemicals

FTY720 (C/N 10006292) and FTY720-P (C/N 10008639) was purchased from the Cayman Chemical Company (Ann Arbor, MI). TNF was from R&D Systems (Minneapolis, MN). Unless otherwise specified, all chemicals used in this study were purchased from Sigma-Aldrich (St. Louis, MO).

### Cell culture

The human alveolar epithelial cell line (A549) was cultured in Ham’s F-12K (Kaighn’s) medium (Invitrogen, Carlsbad, CA) supplemented with penicillin (100 U/ml), streptomycin (100 μg/ml), and fetal calf serum (10%)^4^,^13^. All experiments were performed after an overnight serum-starvation period (14–16 h) in Ham’s F-12K supplemented with sterile BSA (0.1%) and stimulated with 4 ng/ml TNF in accordance with conditions originally reported by Cornell *et al.*^13^. A minimum of three experimental replicates performed on separate days were performed for each experiment.

### PP2A activity assay

PP2A activity was determined using the PP2A immunoprecipitation and phosphatase assay kit (Merck Millipore, Darmstadt, Germany) according to the manufacturer’s instructions.

### Real-time RT-PCR

Total RNA was extracted using the RNeasy Mini Kit (Qiagen Australia, Doncaster, VIC, Australia) and reverse transcription performed using the RevertAid First strand cDNA Synthesis kit (Fermentas Life Sciences, Hanover, MD) according to the manufacturer’s protocol. IL-6, IL-8 and COX-2 mRNA levels were measured using real-time RT-PCR on an ABI Prism 7500 (Applied Biosystems, Foster City, CA). IL-8 (Hs00174103_m1), IL-6 (Hs00174131_m1) and COX-2 (Hs0015133_m1) TaqMan gene expression assays and the eukaryotic 18S rRNA endogenous control probe (Applied Biosystems) were used and subjected to the following cycle parameters: 50 °C for 2 min, 1 cycle; 95 °C for 10 min, 1 cycle; 95 °C for 15 s, 60 °C for 1 min, 40 cycles and mRNA expression (fold increase) quantified by delta delta Ct calculations.

### ELISA

IL-6 and IL-8 ELISAs were performed according to the manufacturer’s instructions (BD Biosciences Pharmingen, San Diego, CA).

### Western blotting

Western blotting in whole cell lysates was performed using rabbit polyclonal antibodies against IκB-α (C-21: Santa Cruz Biotechnology, Santa Cruz, CA) and α-tubulin was used as the loading control (mouse monoclonal IgG_1_, DM1A: Santa Cruz Biotechnology). Primary antibodies were detected with goat anti-mouse or anti-rabbit HRP–conjugated secondary antibodies (Cell Signaling Technology) and visualized by enhanced chemiluminescence (PerkinElmer, Wellesley, MA).

### PGE_2_ assay

PGE_2_ was measured by enzyme immunoassay (Prostaglandin E_2_ EIA 514010: Cayman Chemical Company) according to the manufacturer’s instructions.

### Statistical analysis

Statistical analysis was performed using two-way ANOVA followed by Bonferroni’s post-test or the Student’s unpaired *t* test. *P* values <0.05 were sufficient to reject the null hypothesis for all analyses. Data are mean + SEM of n ≥ 3 independent replicates.

## Additional Information

**How to cite this article**: Rahman, M. M. *et al.* The phosphorylated form of FTY720 activates PP2A, represses inflammation and is devoid of S1P agonism in A549 lung epithelial cells. *Sci. Rep.* **6**, 37297; doi: 10.1038/srep37297 (2016).

**Publisher’s note**: Springer Nature remains neutral with regard to jurisdictional claims in published maps and institutional affiliations.
